# Supplementary material for: Simultaneous perception of prosthetic and natural vision in AMD patients
Source: Nat Commun. 2022 Jan 26;13:513. doi: 10.1038/s41467-022-28125-x (PMC8792035; doi:10.1038/s41467-022-28125-x)
Supplement: Supplementary file 2 — Description of Additional Supplementary Files [file 41467_2022_28125_MOESM2_ESM.docx]

Description of Additional Supplementary Files

**Title: Supplementary Video 1**.

Description: Illustration of the patient’s letter recognition using PRIMA-2 glasses at x4 magnification and a contrast reversal.

**Title: Supplementary Video 2**.

Description: Illustration of the patient’s reading ability with PRIMA-2 glasses at x4 magnification and a contrast reversal.

**Title: Supplementary Video 3**.

Description: Control experiment for reading the same word as in S2, but without PRIMA-2 glasses.

**Title: Supplementary Video 4**.

Description: Simultaneous detection of the bars using prosthetic and natural vision in both eyes.

**Title: Supplementary Video 5**.

Description: Simultaneous detection of the bars using prosthetic and natural vision in the same eye.
