# Supplementary material for: Simultaneous perception of prosthetic and natural vision in AMD patients
Source: Nat Commun. 2022 Jan 26;13:513. doi: 10.1038/s41467-022-28125-x (PMC8792035; doi:10.1038/s41467-022-28125-x)
Supplement: Supplementary file 8 — Reporting Summary [file 41467_2022_28125_MOESM8_ESM.pdf]

nature research

Corresponding author(s): Daniel Palanker

Last updated by author(s): November 4, 2021

Reporting Summary

Nature Research wishes to improve the reproducibility of the work that we publish. This form provides structure for consistency and transparency in reporting. For further information on Nature Research policies, see our Editorial Policies and the Editorial Policy Checklist.

Statistics

For all statistical analyses, confirm that the following items are present in the figure legend, table legend, main text, or Methods section.

n/a	Confirmed	
☐	☒	The exact sample size (*n*) for each experimental group/condition, given as a discrete number and unit of measurement
☐	☒	A statement on whether measurements were taken from distinct samples or whether the same sample was measured repeatedly
☐	☒	The statistical test(s) used AND whether they are one- or two-sided<br>*Only common tests should be described solely by name; describe more complex techniques in the Methods section.*
☒	☐	A description of all covariates tested
☒	☐	A description of any assumptions or corrections, such as tests of normality and adjustment for multiple comparisons
☒	☐	A full description of the statistical parameters including central tendency (e.g. means) or other basic estimates (e.g. regression coefficient) AND variation (e.g. standard deviation) or associated estimates of uncertainty (e.g. confidence intervals)
☒	☐	For null hypothesis testing, the test statistic (e.g. *F*, *t*, *r*) with confidence intervals, effect sizes, degrees of freedom and *P* value noted<br>*Give P values as exact values whenever suitable.*
☒	☐	For Bayesian analysis, information on the choice of priors and Markov chain Monte Carlo settings
☒	☐	For hierarchical and complex designs, identification of the appropriate level for tests and full reporting of outcomes
☒	☐	Estimates of effect sizes (e.g. Cohen's *d*, Pearson's *r*), indicating how they were calculated

Our web collection on statistics for biologists contains articles on many of the points above.

Software and code

Policy information about availability of computer code

Data collection	Freiburg Visual Acuity Test (FrACT) software, version 3.10.3a
Data analysis	Freiburg Visual Acuity Test (FrACT) software, version 3.10.3a

For manuscripts utilizing custom algorithms or software that are central to the research but not yet described in published literature, software must be made available to editors and reviewers. We strongly encourage code deposition in a community repository (e.g. GitHub). See the Nature Research guidelines for submitting code & software for further information.

Data

Policy information about availability of data

All manuscripts must include a data availability statement. This statement should provide the following information, where applicable:

- Accession codes, unique identifiers, or web links for publicly available datasets
- A list of figures that have associated raw data
- A description of any restrictions on data availability

The authors declare that all the data supporting the findings of this study are available within the paper and its supplementary information files. Any additional information, including the approved study protocols and data analysis software, are available from the corresponding author upon a reasonable request.

Field-specific reporting

Life sciences study design

All studies must disclose on these points even when the disclosure is negative.

Sample size	Five patients - the number approved for this feasibility study by the Agence Nationale de Sécurité du Médicament et des Produits de Santé in France (ASNM) for initial assessment of the safety of the implantation and elicitation of the visual response.
Data exclusions	One patient had an implant inside the choroid, and therefore had no prosthetic acuity. All other four patients had an implant in subretinal space, and had prosthetic acuity. Therefore, the first patient was excluded from the average acuity calculation.
Replication	Visual acuity testing was performed three times on three different days. At each day, 24 trials were performed twice, and the mean of these two runs was considered the daily result. The final result was defined as the median of the three daily measurements. All attempts of replication were successful.
Randomization	All the patients were tested in one group.
Blinding	Blinding was not possible because the augmented reality glasses have asymmetric design, i.e. only the study eye has a NIR display, as shown in Figure 1 and 2, while the fellow eye is open.

Reporting for specific materials, systems and methods

We require information from authors about some types of materials, experimental systems and methods used in many studies. Here, indicate whether each material, system or method listed is relevant to your study. If you are not sure if a list item applies to your research, read the appropriate section before selecting a response.

Materials & experimental systems

n/a	Involved in the study	
☒	☐	Antibodies
☒	☐	Eukaryotic cell lines
☒	☐	Palaeontology and archaeology
☒	☐	Animals and other organisms
☐	☒	Human research participants
☐	☒	Clinical data
☒	☐	Dual use research of concern

Methods

n/a	Involved in the study	
☒	☐	ChIP-seq
☒	☐	Flow cytometry
☒	☐	MRI-based neuroimaging

Human research participants

Policy information about studies involving human research participants

Population characteristics	Study participants (2 male and 3 female) were above 60 years of age and had advanced atrophic AMD with an atrophic zone of at least 3 optic disc diameters and best corrected visual acuity of ≤20/400 in the worse-seeing study eye; no foveal light perception (absolute scotoma) but visual perception in the periphery, with preferred retinal locus determined by micro-perimetry; absence of photoreceptors and presence of the inner retina in the atrophic area as confirmed by optical coherence tomography (OCT); absence of choroidal neovascularization verified by retinal angiography. All other ocular and general pathologies that could contribute to the low visual acuity were excluded.
Recruitment	Patients from the Foundation A Rothschild Hospital (Paris, France) and from the Clinical Investigation Center of Quinze-Vingts National Eye Hospital (Paris, France) that met the clinical inclusion criteria were presented with the information letter (in French). Patients who expressed an interest in participating in the study were provided a written informed consent. A more detailed assessment of the patients for confirmation of the match to the inclusion and exclusion criteria was done after the informed consent was signed. Implantation took place at the Foundation A Rothschild Hospital (Paris, France). The patients' rehabilitation and visual function assessment were carried out at the Clinical Investigation Center of Quinze-Vingts National Eye Hospital (Paris, France).<br>In addition to the five implanted subjects, the following three other subjects signed the patient informed consent form, but were not implanted:<br>- One subject was enrolled but withdrawn by the investigator due to unrealistic expectations.<br>- One subject withdrew the consent prior to implantation.<br>- One patient signed the patient informed consent but after that it was detected that not all inclusion and exclusion criteria were fulfilled, thus the subject was withdrawn.<br>Regarding the self-selection bias: Usually, patients enrolling in clinical trials are better informed, and more motivated than average, but this applies to all clinical trials.
Ethics oversight	The study adhered to the Declaration of Helsinki and received the ethics committee approval from the Comité de Protection des Personnes Ile de France II and approval by the Agence Nationale de Sécurité du Médicament et des Produits de Santé in France.

Note that full information on the approval of the study protocol must also be provided in the manuscript.

Clinical data

Policy information about clinical studies

All manuscripts should comply with the ICMJE guidelines for publication of clinical research and a completed CONSORT checklist must be included with all submissions.

Clinical trial registration	NCT03333954
Study protocol	The study protocol is not public, but can be provided to the editors, if necessary.
Data collection	Implantation took place at the Foundation A Rothschild Hospital (Paris, France). The patients' rehabilitation and visual function assessment were carried out at the Clinical Investigation Center of Quinze-Vingts National Eye Hospital (Paris, France).<br>Recruiting: Q4 2017 - Q2 2018. Data collection: Q4 2017 - Q4 2020.
Outcomes	1) Visual acuity:<br>Visual acuity was assessed using a computer-generated Landolt C in 4 different orientations (gap at the top, bottom, right or left), so that a random response corresponds to 25% accuracy. The threshold optotype size was defined as the proper symbol recognition with at least 62.5% accuracy. To minimize the number of presentations, the study was conducted using the method of the Freiburg Acuity and Contrast Test (FrACT) test14. In this protocol, a single Landolt C is presented in a fixed central position on a display, and the best Parameter Estimation by Sequential Testing (best PEST) procedure16 was used to estimate the VA. Since it is an adaptive test, the number of times that each optotype is presented varies depending on the patient responses. The test was performed three times on three different days. At each day, 24 trials were performed twice, and the mean of these two runs was considered the daily result. The final result was defined as the median of the three daily measurements. To mimic the crowding effect of the letters in vision charts, Landolt C was presented with a frame around it.<br>2) Background illumination<br>To provide a uniform and controllable background illumination, a 71 cm-wide Samsung U28E590D LCD screen has been used. Subjects were placed 40 cm in front of a screen, where a homogeneous white illumination at 16 levels was presented: 256, 181, 128, 90.5, 64, 45.3, 32, 22.6, 16, 11.3, 8, 5.7, 4, 2.8, 2, 1.4 cd/m², while room lights were turned off.<br>The best-PEST (Parameter Estimation by Sequential Testing) method16 was used to determine the maximum luminance which still allowed identifying the prosthetic patterns (Landolt C optotypes with four different orientations) with accuracy exceeding 62.5% using the following parameters: 20 iterations, 0.25 false positive rate, 0 false negative rate, and sigmoid slope of 0.5. The last value of the best-PEST was used as the resulting threshold luminance. Data for the three patients with a subretinal implant are presented, but not for subject #1 since this subject with intra-choroidal chip placement could not recognize Landolt C. Prosthetic patterns were presented at maximum brightness: 3.5 mW/mm2 of NIR irradiance with 9.8 ms pulse duration and 30 Hz repetition rate. The patterns were presented for up to 30 seconds, with 10 seconds break between the stimuli.<br>3) Simultaneous perception of prosthetic and natural vision:<br>Simultaneous perception of prosthetic and natural vision<br>A green bar is displayed on an LCD screen placed at 40 cm in front of the subject in one of the four possible orientations (vertical, horizontal, 45° diagonal from the upper left, 45° diagonal from the lower left). Simultaneously a bar is also projected in Artificial Pattern Mode (APM) on the PRIMA Glasses in one of the four orientations (Figure 3b). The width of the bars corresponds to 0.4 mm on the retina (4 implant pixels). The bar orientation on LCD screen is random, but 50% of the times orientation of the bar displayed on the glasses matches the bar orientation on LCD. The subject is expected to see the bar displayed on the LCD screen with the natural peripheral vision and report it as green, and the bar projected by the PRIMA Glasses with prosthetic vision, perceived as white. At each repetition, the subject is asked about orientation of each bar individually.<br>A total of 48 bar pairs were presented (24 presentations with the fellow eye open and 24 presentation with the fellow eye closed), each for a duration of up to 20 seconds. The subjects were not allowed to move their head – only the eyes.
